# Getting the right balance: insole design alters the static balance of people with diabetes and neuropathy

**DOI:** 10.1186/s13047-016-0172-3

**Published:** 2016-10-05

**Authors:** Joanne Paton, Sam Glasser, Richard Collings, Jon Marsden

**Affiliations:** 1 School of Health Professions, Peninsula Allied Health Centre, Plymouth University, Marjon Campus, Derrifod Road, Plymouth, PL6 8BH UK; 2 Podiatry Service Torbay and South Devon Health and Care NHS Trust, Castle Circus Health Centre, Abbey Road, Torquay, TQ2 5YH UK

**Keywords:** Diabetic, neuropathy, insoles, balance

## Abstract

**Background:**

Over 1 in 3 older people with diabetes sustain a fall each year. Postural instability has been identified as independent risk factor for falls within people with Diabetic Peripheral Neuropathy (DPN). People with DPN, at increased risk of falls, are routinely required to wear offloading insoles, yet the impact of these insoles on postural stability and postural control is unknown. The aim of this study was to evaluate the effect of a standard offloading insole and its constituent parts on the balance in people with DPN.

**Methods:**

A random sample of 50 patients with DPN were observed standing for 3 × 30 s, and stepping in response to a light, under five conditions presented in a random order; as defined by a computer program; 1) no insole, 2) standard diabetic: a standard offloading insole made from EVA/poron®, and three other insoles with one design component systematically altered 3) flat: diabetic offloading insole with arch fill removed, 4) low resilient memory: diabetic offloading insole with the cover substituted with low resilience memory V9, 5) textured: diabetic offloading insole with a textured PVC surface added (Algeos Ltd). After each condition participants self-rated perceived steadiness.

**Results:**

Insole design effected static balance and balance perception, but not stepping reaction time in people with DPN. The diabetic and memory shaped insoles (with arch fill) significantly increased centre of pressure velocity (14 %, *P* = 0.006), (13 %, *P* = 0.001), and path length (14 %, *P* = 0.006), (13 %, *P* = 001), when compared to the no insole condition. The textured shaped and flat soft insole had no effect on static balance when compared to the no insole condition (*P* > 0.05).

**Conclusion:**

Insoles have an effect on static balance but not stepping reaction time. This effect is independent of neuropathy severity. The addition of a textured cover seems to counter the negative effect of an arch fill, even in participants with severe sensation loss. Static balance is unaffected by material softness or resilience. Current best practice of providing offloading insoles, with arch fill, to increase contact area and reduce peak pressure could be making people more unstable. Whilst flat, soft insoles maybe the preferable design option for those with poor balance. There is a need to develop an offloading insole that can reduce diabetic foot ulcer risk, without compromising balance.

## Background

Over 1 in 3 older people with diabetes sustain a fall each year [1], increasing to 1 in 2 for those with prior foot ulceration [2]. The risk of experiencing a fall is heightened in the presence of diabetic peripheral neuropathy [3]. People with diabetes and neuropathy are 15 times more likely to report an injurious fall than those without neuropathy [4]. Neuropathy affects approximately half of people who have had diabetes for 20 years [4]. Clinical manifestations of neuropathy include cutaneous sensation loss, lower limb muscle weakness and reduced joint motion [5, 6]. The functional impact of these changes is gait dysfunction and poor balance due to a loss of cutaneous receptors and proprioceptive information [7, 8]. Postural instability has been identified as an independent risk factor for falls within the diabetic neuropathic population [7].

Despite insole and footwear provision being a well-established element of the multidisciplinary neuropathic foot ulcer prevention strategy, the influence of this intervention on balance and falls risk appears to have been largely overlooked. Offloading insoles are often total contact devices, constructed from thick and soft smooth foam materials intended to cushion and protect the plantar foot from mechanical stress and reduce ulcer risk [9]. Whilst the arch fill total contact element of the insole has the potential to aid mechanical stability and optimise the number of cutaneous mechanoreceptors receiving afferent surface information, other design features, (such as the thick soft foam covering materials) common to diabetic insoles may have a detrimental effect on balance. To date the majority of studies investigating the effects of covering materials on balance have focused attention on healthy elderly participants [10, 11]. It is unclear if the findings of such studies have relevance to the diabetic neuropathic population.

The results of a small number of laboratory studies using healthy elderly participants have implied that standing on smooth, soft thick materials, such as that prescribed for diabetic individuals with neuropathy, may exacerbate cutaneous sensation loss by ‘shielding’ the foot from sensation, reducing cutaneous input and decreasing balance and increasing falls risk [10, 11]. Thus it is recommended that people who have suffered a fall wear a safe stable style of shoe consisting of a firm, not cushioned, sole to maximise plantar cutaneous sensory awareness [12]. Others have implied that in people without diabetic neuropathy standing on a textured or raised surface could heighten cutaneous stimulation and increase neural feedback from cutaneous receptors [13, 14].

People with diabetes and neuropathy are routinely required to wear offloading insoles incorporating an arch fill and smooth soft top cover to reduce foot ulcer risk. This type of insole has the potential to influence balance, yet its impact on balance in the diabetic population at increased risk of falls is undetermined. The primary aim of this study is to investigate the effect of offloading insoles on balance in people with diabetes and neuropathy. The secondary purpose of this study is to systematically manipulate each design component common to insoles for people with diabetes to investigate the impact of each component on balance.

## Methods

Ethical approval was granted by the Cornwall and Plymouth Research Ethics Committee (REC number: 13/SW/0334). All participants gave written informed consent prior to data collection.

### Participants

From March 2014 to March 2015, a total of 420 people with diabetes at increased or high risk of foot ulcer, were selected at random by a third party (not a member of the research team) using a computer programme from the Plymouth Community Healthcare Podiatry Service Database and invited by post to be assessed for eligibility. In total, 50 were recruited into the study. Participants were considered eligible if they had a self-reported diagnosis of diabetes mellitus, had been identified as having diabetes on the database, had insensate or diminished sensation, defined as insensitivity of a 10 g monofilament at one or more sites in the following locations: hallux, 1st, 3rd, and 5th metatarsal heads [15], were able to stand unaided for 30 s with eyes closed and step up onto a step, were living in the community. Participants were excluded from the study if they had undergone a major amputation of the lower limb, presented with an active foot ulceration or Charcot arthropathy, had been prescribed medication known to affect balance within the last 6 months, were unable to follow simple instructions, self-reported other neurological or vestibular disorder or a history of episodes of dizziness, suffered from a condition known to impair cognitive function.

### Insole conditions

Each participant wore standardised 20 denier hosiery and CHAUSSURES pulman ® classic house shoes. A highly adjustable Velcro fastening house shoe made from a textile material called RAZETTI ®.

**Fig. 1:**
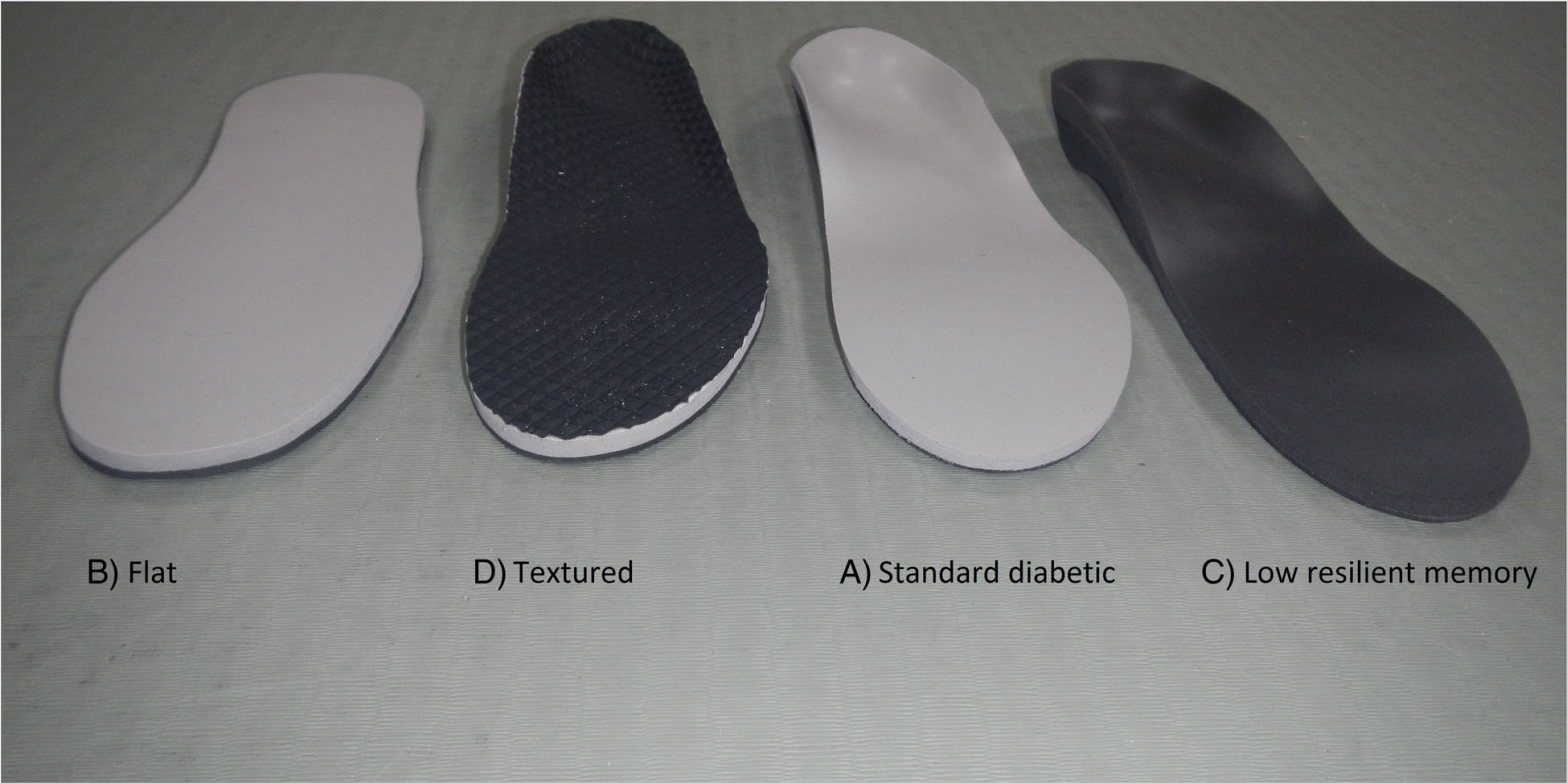
The four insole test conditions. **b** Flat: diabetic offloading insole with arch fill removed; **d** Textured: diabetic offloading insole with a textured PVC surface added (Algeos Ltd). **a** Standard diabetic: A standard offloading insole made from a prefabricated 3 mm full length medium EVA contoured shell (slimflex algeos Ltd) and covered in 6 mm of poron® (**c**) Low resilient memory: diabetic offloading insole with the cover substituted with low resilience memory V9

Participants were fitted with four different insoles and a no insole condition, Fig. 1; A) Standard diabetic: the diabetic offloading insole (unmodified) consisting of a prefabricated 3 mm full length medium EVA contoured shell (slimflex Algeos Ltd) covered in 6 mm of poron®, designed to represent the standard insole traditionally issued to people with diabetes and neuropathy to reduce foot ulcer risk. Three other insole conditions had one element of the standard diabetic insole systematically altered; B) Flat: Standard offloading insole with the arch fill removed constructed by replacing the moulded shell with a flat 3 mm medium EVA base (the 6 mm poron cover remained unchanged); C) Low resilient memory: Diabetic insole with a low resilience memory cover. Constructed by replacing the high resilient 6 mm poron® cover with a low resilient, slow return 6 mm memory V9 cover (the moulded medium EVA base unchanged); D). Textured: Diabetic offloading insole with textured PVC cover. Constructed by covering the smooth 6 mm poron top surface with Tec black, a textured PCV, (Algeos Ltd), (the moulded 3 mm medium EVA base 6 mm poron cover remained the same).

### Procedure

This study was a repeated-measures experimental design; all within-subject measures were performed by the JP, in a single session, at the Human Movement Laboratory, Plymouth University. Severity of cutaneous sensation loss was first measured using vibration perception threshold at three sites across each foot and lower limb (neurothesiometer). The selected sites included the apex of the hallux, the medial aspect of the first metatarsophalangeal joint and the lateral malleolus. Then standing balance, step reaction time, and participant perceived stability were assessed under five test conditions. The five insole conditions were presented in a random order defined by a computer program. Participants rested in a sitting position for a minimum of 2 min between conditions to prevent fatigue and allow the primary investigator (JP) to fit the next insole and fasten the house shoe.

### Standing balance

It is commonly accepted that body oscillations during normal quiet standing are indicative of postural stability [12]. Smaller amplitudes and lower speeds of body sway are considered representative of better postural stability in terms of less effort needed to maintain posture. The portable force platform (F-Scan) was used to collect COP data as a measure of static balance. This equipment was selected because it is portable, easy to use and has a low time burden. It could therefore be considered suitable for multiple patient use within the hospital setting.

Participants were weighed and the pressure mat calibrated (F-scan version 7: Tekscan Ltd South Boston Massachusetts). Then three 30-s trials were recorded for each test condition at a sampling frequency 100Hz.

Participants were instructed to stand “as still as possible” on the pressure mat, with their eyes closed and their feet parallel 4 cm apart. Vision was constrained to limit visual sensory feedback and optimise reliance on somatosensory receptors (proprioceptors and mechanoreceptors). Foot position was constrained to minimise variability between participants and across conditions due to changes in stance width and foot angle.

Movement of the centre of pressure (COP) was exported into MATLAB and filtered using a 6th order 5Hz butterworth low pass filter. The first 5 s of each trial was excluded to give time for participants to stabilise. Total velocity of COP data and velocity of COP in the medial/lateral (ML), anterior/posterior (AP) directions were calculated, likewise total path length of COP data and path length in the ML and AP directions were calculated.

The mean of three trials for each parameter was taken forward for statistical analysis.

### Perceived stability

Once the standing balance test had been completed for each insole condition participants were asked to rate on a scale of 1 to 10 how stable they felt whilst undertaking the test, if 10 indicated most stable imaginable.

### Step reaction time

A dynamic measure was selected as an outcome for this study to reflect the fact that the majority of falls occur while people undertake dynamic activities. More specifically step reaction time was considered of particular relevance to our target patient group because people with diabetes and neuropathy interviewed for our previous work described catching a toe whilst stepping up a change in level as a common event behind their fall [16]. In addition, others have used a ‘timed stepping test’ to prospectively classify the falls status of elderly community dwellers [17].

Participants underwent a dynamic evaluation of posture control, whilst negotiating change in level. The F-scan in-shoe pressure measurement system set at 100Hz sampling frequency was used to capture stepping reaction time. Before each data collection session each participant was fitted with a pair of in-shoe pressure sensors placed beneath the test insole. Once fitted each pair of sensors were calibrated against body weight. Once positioned in front of the step participants were encouraged to practice stepping onto the step. Participants were then asked to respond to a warning followed by a response light by stepping as quickly as possible leading with the side indicated (left or right) onto a step raised by 15 cm. The time taken to complete the stepping task was analysed in the following stages; reaction time, weight shift time, step time one, step time two and total movement time (adapted from Bruer and Burns et al 2000 [17]). The recording was triggered when the response light activated. During data collection the identity of the lead foot (left or right) was alternated at random to prevent participants pre-empting the lead foot and beginning weight shift before the signal.

The quicker of two attempts leading with the same foot were taken forward for analysis.

### Statistical analysis

Sample size was based on COP velocity values for older people with eyes closed sourced from Losa Iglesias and colleagues 2012 [18]. They found a difference between 2.51 mm/s (SD1.60) for a soft insole, and 2.87 mm/s (SD1.81) when barefoot (giving a pooled standard deviation of 1.76). Using this data an effect size of 0.20 was determined and assumed for this study. A study sample size of 50 provided 80 % power at *p* = 0.05, for repeated measures ANOVA of one sample containing 5 continuous response variables.

All statistical analysis was carried out using SPSS Version 21. (IBM Armonk NY USA). The sway and step data was not in violation of any of the assumptions that apply to parametric techniques. Therefore one-way repeated measures analysis of variance was used to assess the effect of the insoles on static balance and stepping reaction time. Bonferroni adjustment was applied to all pairwise comparisons. The effect of insoles on perceived stability was investigated using the Friedman Test. Further exploratory post-hoc sub-group analysis investigated whether neuropathy severity modified the effect of each insole condition on static balance.

### Participant characteristics

The of participant group included 43 with type 2 diabetes and 7 with type 1, with an average age of 71 years (SD8) height of 1.74 m (SD0.11), weight of 97.6 kg (SD20.05), body mass index of 32.43 (SD16.31), and a sensory perception threshold of 37.32 V (SD9.966), with 38 being male and 12 female.

## Results

### Comparison of insoles

**Fig. 2:**
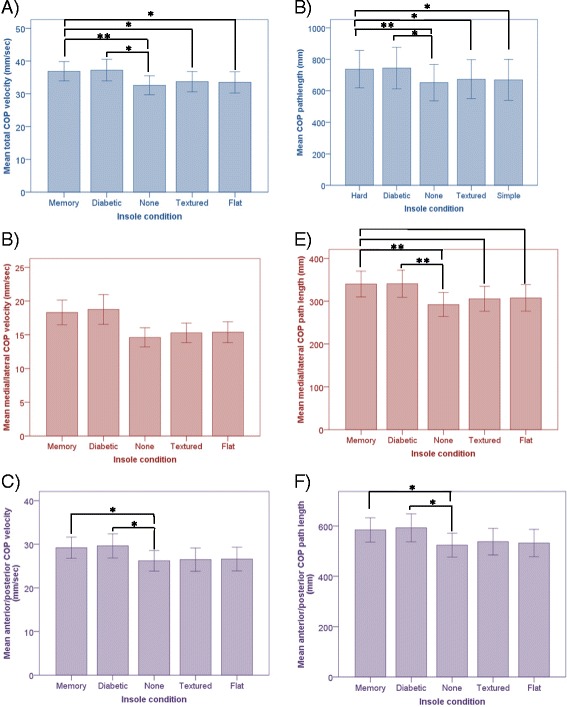
Estimated means +/- standard errors during static, standing eyes closed whilst wearing each insole condition. **a** Total COP velocity, **b** Medial/lateral COP velocity, **c** Anterior/posterior COP velocity, **d** Total COP path length, **e** Medial/lateral COP path length, **f** Anterior/posterior COP path length. Significant pairwise comparisons: *P < 0.05, **P < 0.001

A comparison of the effect of each insole condition (low resilient memory, standard diabetic, textured, flat and none) on measures of static balance and stepping reaction showed there was no significant effect for insole condition on step reaction time (Table 1). There was a significant effect for insole condition for all parameters of static balance except medial/lateral COP velocity, (Table 2 and Fig. 2).

**Table 1:** ANOVA results comparing the effect of each insole condition on stepping reaction time (seconds)

	Insole condition										*P* value
	No insole		Diabetic		Flat		Memory		Textured		
	Mean (SD)	95 % CI	Mean (SD)	95 % CI	Mean (SD)	95 % CI	Mean (SD)	95 % CI	Mean (SD)	95 % CI	
RT	1.224 (0.294)	1.139-1.308	1.276 (0.383)	1.166-1.386	1.251 (0.217)	1.178-1.302	1.255 (0.392)	1.143-1.368	1.251 (0.287)	1.169-1.334	F = 0. 459
											*P* = 0.7
											Eta = 0.039
WS	0.573 (0.211)	0.513-0.633	0.538 (0.152)	0.495-0.582	0.577 (0.152)	0.534-0.620	0.572 (0.216)	0.511-0.634	0.511 (0.174)	0.461-0.560	F = 2.709
											*P* = 0.042
St1	1.774 (0.413)	1.657-1.892	1.834 (0.400)	1.721-1.949	1.863 (0.369)	1.758-1.968	1.8429 (0.426)	1.722-1.964	1.884 (0.468)	1.751-2.018	F = 1.134
											*P* = 0.352
											Eta = 0.09
St2	0.448 (0.140)	0.408-0.488	0.463 (0.099)	0.436-0.492	0.428 (0.146)	0.387 (0.470)	0.461 (0.127)	0.425-0.498	0.451 (0.188)	0.398-0.505	F = 1.180
											*P* = 0.332
											0.093
TM	2.086 (0.569)	1.924-2.248	2.063 (0.438)	1.938-2.188	2.168 (0.470)	2.035-2.302	2.123 (0.493)	1.983-2.264	2.133 (0.611)	1.960-2.308	F = 0.767
											*P* = 0.552
											Eta = 0.063

*Memory* low resilient memory insole, *Diabetic* standard diabetic offloading insole, *Textured* textured insole, *Flat* flat insole, *RT* reaction time, *WS* weight shift, *St1* step one, *St2* step two, *TM* total movement, *SD* standard deviation, *CI* confidence interval

**Table 2:** ANOVA results comparing the effect of each insole condition on static balance

	Insole Condition										*P* value
	No insole		Diabetic		Flat		Memory		Textured		
	Mean (SD)	95 % CI	Mean (SD)	95 % CI	Mean (SD)	95 % CI	Mean (SD)	95 % CI	Mean (SD)	95 % CI	
Total COP velocity	32.350 (19.698)	26.735-39.949	36.952 (22.522)	30.551-43.353	33.267 (22.070)	26.995-39.539	36.572 (20.197)	30.833- 42.313	33.472 (21.049)	27.499-39.455	F = 5.769
											*P* = 0.001
											Eta = 0.334
Medial/lateral COP velocity	14.514 (9.416)	11.782 –17.247	18.692 (14.968)	14.438 – 22.946	15.284 (10.580)	12.277-18.291	18.198 (12.490)	14.647 – 21.748	15.174 (9.927)	12.353-17.995	F = 2.310
											*P* = 0.072
											Eta = 0.167
Ant/post COP velocity	26.009 (16.051)	21.447 – 30.571	29.431 (18.876)	24.067 – 34.797	26.467 (18.432)	21.232 – 31.704	28.977 (16.415)	24.312 - 33.642	26.327 (18.186)	21.158 – 31.496	F = 4.346
											*P* = 0.005
											Eta = 0.274
Total COP path length	647.015 (393.96)	535.05-758.98	739.041 (450.44)	611.02-867.05	665.342 (441.40)	539.89-790.78	732.455 (403.95)	616.654 –846.258	669.457 (420.99)	549.81-789.10	F = 5.769
											*P* = 0.001
											Eta = 0.334
Medial/lateral COP path length	290.281 (192.29)	235.63 -344.93	338.272 (215.33)	227.07– 399.47	305.680 (221.61)	245.54-365.92	337.576 (204.91)	279.341-395.812	303.482 (198.54)	247.05 -359.90	F = 6.936
											*P* < 0.001
											Eta = 0.376
Ant/post COP path length	520.181 (321.02)	428.94-611.41	588.636 (377.53)	481.34– 695.93	529.358 (368.46)	424.64– 634.07	579.545 (328.30)	486.243 – 672.849	534.593 (357.32)	433.02 -636.14	F = 3.393
											*P* = 0.008
											Eta = 0.255

*Memory* low resilient memory insole, *Diabetic* standard diabetic offloading insole. *Textured* textured insole. *Flat* flat insole, *COP* centre of pressure, *Ant/post* anterior posterior, *SD* standard deviation, *CI* confidence interval

There was no significant difference in any parameter of COP velocity or path length when standard diabetic and low resilience memory insoles were compared. Likewise, no significant difference in any parameter of COP velocity and path length was found when no insole, flat insole and textured insole conditions were compared.

Total COP velocity, total COP path length and medial/lateral COP path length was significantly greater when participants wore the low resilience memory insoles compared to no insole, textured insole and flat insole (Table 3). This trend was also apparent for anterior/posterior COP velocity and path length but only reached a level of significance when low resilience memory insoles were compared to no insoles (Fig. 2).

**Table 3:** Pairwise comparisons of insole conditions with statistical significance *p* < 0.5

Pairwise comparisons of insole conditions	Mean Difference (SE)	Significance	95 % CI values
Total COP velocity, mm/s			
Memory and none	4.222 (0.976)	0.001	1.352-7.092
Memory and textured	3.1 (1.026)	0.04	0.084-6.115
Memory and flat	3.306 (1.053)	0.029	0.210-6.401
Diabetic and none	4.601 (1.249)	0.006	0.930-8.272
Anterior/posterior COP velocity, mm/s			
Memory and none	2.968 (0.842)	0.009	0.494-5.443
Diabetic and none	3.423 (1.159)	0.048	0.016-6.830
Total COP path length, mm			
Memory and none	84.440 (19.526)	0.001	27.038-141.843
Memory and textured	61.998 (20.515)	0.04	01.688-122.308
Memory and flat	66.113 (21.062)	0.029	04.198-128.029
Diabetic and none	92.026 (24.976)	0.006	18.604-165.448
Medial/Lateral COP path length, mm			
Memory and none	47.295 (10.857)	0.001	15.379-79.212
Memory and textured	34.094 (10.308)	0.018	03.731-64.398
Memory and flat	31.896 (9.077)	0.01	05.212-58.579
Diabetic and none	47.992 (11.167)	0.001	15.163-80.820
Anterior/posterior COP path length, mm			
Memory and none	59.364 (16.836)	0.009	9.871-108.857
Diabetic and none	68.455 (23.180)	0.048	0.310-136.599

*Memory* low resilient memory insole, *Diabetic* standard diabetic offloading insole. *Textured* textured insole, *Flat* flat insole, *SE* standard error, *CI* confidence intervals, *COP* centre of pressure

Total COP velocity, anterior/posterior COP velocity and total path length, medial/lateral path length, and anterior/posterior path length was significantly greater when participants wore the standard diabetic insole compared to the no insole condition (Table 3). A similar pattern was seen when the standard diabetic insole was compared to the textured and flat insole conditions but a level of significance was not met (Fig. 2).

### Perception of stability

Comparing the Mean Ranks for the five sets of scores it appears that there was a decrease in the rank score for perception of stability when the low resilience memory (2.73) and standard diabetic insoles (2.83) were compared to the other conditions (no insole = 3.34, flat = 3.07, textured = 3.02). This finding however did not reach a level of statistical significance (*p* > 0.05). Group median values were six (IQR = 2.75) out of ten for the low resilient memory insole condition and seven (IQR = 2 none, flat, textured and 3 standard diabetic) for all other conditions.

## Discussion

The primary aim of this study was to investigate the effect of total contact standard diabetic insoles on balance in people with diabetes and neuropathy. We found that the offloading standard diabetic total contact insole with arch fill and smooth, cushioned top cover, (similar to that commonly used to reduce foot ulcer risk in people with diabetic peripheral neuropathy) reduced static balance (increased postural sway velocity) in participants with diabetes and neuropathy. However insole type had no effect on stepping reaction time.

The findings might suggest that insole design could artificially alter (dampen or enhance) any remaining somatosensory awareness, that contributes to the maintenance of postural stability, in people with diabetes and neuropathy. However variations in insole design may not influence the reaction time of any rapid voluntary corrective balance response. Stepping reaction time is in part dependent on the planning, transmission and response of the descending motor command and confounded by a number of contributing variables including visual acuity, level of motivation and concentration, amount of practice, decision making ability and cognitive function. Moreover reaction time has been found by others to be independent of body sway [19]. Thus it is unsurprising to find that stepping reaction time was not a sensitive measure of the afferent feedback provided by insoles in this study.

The second aim of the study was to systematically manipulate each design component common to insoles for people with diabetes to investigate which component altered balance in people with diabetes and neuropathy. Specifically three insole design components were manipulated; 1. The total contact insole with arch fill was made flat; 2. The resilient, fast return top cover material was swapped for a material of low resilience, a slow return memory material 3. The smooth top surface was covered with a textured material.

### The effect of the insole profile on standing balance

Static balance was significantly worse when participants wore the standard diabetic and low resilient memory insoles compared to the flat and no insole conditions. For example total COP path length increased by a mean of 14 % (absolute mean 92 mm) when the standard diabetic insole was compared to the no insole condition. The percent difference in static balance was even greater in the medial/lateral direction when the standard diabetic insole was worn; increasing by a mean of 17 % (absolute mean 48 mm). The standard diabetic and flat insoles were constructed from the same materials. The only known difference between the two devices was that the standard diabetic insole had the moulded arch fill and heel cup, whilst the other was flat. It would therefore appear that a moulded arch fill and heel cup impaired the static balance of participants with diabetes and neuropathy.

Only one other study investigated the effect of arch supports on standing balance. Gross and colleagues compared the ability of older people without neuropathy, but with a history of falls, to stand in tandem stance whilst wearing a semi ridged custom made foot orthosis [20]. The authors reported that stance time increased significantly when participants wore the foot orthosis, incorporating an arch support. The discrepancy in findings between studies may be explained either by differences in the insole design or participant characteristics. Both studies used a full length insole design incorporating an arch fill and heel cup, therefore appear that differences in static findings may not relate to the arch fill but instead be attributed differences in insole firmness or participant group. We found that standing balance in people with diabetes was not influenced by insole firmness, and so postulate that people with diabetes and neuropathy display a disease specific response to an arch fill different to those seen in the older population.

Any disease specific alteration in balance stability could be a mechanical or sensory phenomenon. Cutaneous sensation from the plantar mechanoreceptors provides the central nervous system with critical stability information about the proximity of the centre of mass to the base of supports limits and the potential for impending loss of balance [21]. Plantar pressure sensation appears to play an important role [21]. It is reasonable to suppose that the introduction of an arch fill alters the plantar pressure pattern. People with diabetes may detect the proximity of the centre of mass to the limits of the base of support through inversely proportional gross changes in pressure ratios between forefoot and rearfoot. Filling the arch could provide additional confusing sensory midfoot information that makes detection of these proportional changes in regional forefoot and rearfoot pressures more difficult for people with sensory deficit to decipher.

Participants lacking cutaneous perception could be more reliant on foot and ankle joint mechanical stability to retain postural stability. Without arch fill participants maybe more predisposed toward standing with midtarsal, subtalar and ankle joints at end range of motion. We speculate that in this mechanically close packed, more stable position, there is the increased potential to make use of the high load compression and tension mechanoreceptors within the joints that relate information about body schematics. The addition of the arch fill and heel lift could shift these joints out of their close packed position and reduce the loads generated with joints as well as the ability to use high load joint receptors to give proprioceptive information.

### The effect of covering materials on standing balance

Insoles for people with diabetes and neuropathy are often covered with soft thick foam materials designed to cushion and protect the foot from the high loads associated with foot ulcer development. Clinically to meet that objective two types of foam covering materials are routinely selected: 1. Traditionally used open cell foams such as poron®; These are highly resilient materials with excellent, compression set resistance. 2. The more recently developed slow return memory foams such at Memory V9 (Algeos Ltd); This set of materials display low resilience and excellent dampening properties, enabling them to mould to the foot when loaded. Resilience is defined as the amount of energy returned during unloading as a percentage of the amount of energy absorbed during loading [22]. The lower the resilience the greater the dampening or shock attenuation capacity of the material [22]. For this reason memory foams are becoming an increasingly popular insole material choice for people with diabetes. We found that neither the traditional open foam nor the newer slow return memory foams had any effect of standing balance when compared to the no insole condition.

Robbins et al suggested that thick, soft soled footwear intended to provide cushioning to the plantar surface of the foot may reduce dynamic balance through a decrease in foot position awareness in older people [23, 24]. Conversely Lord and colleagues concluded that soft and hard soled shoes made no difference to balance when compared to barefoot in a sample of 42 older women [25]. Likewise, we found that insole firmness had no statistically significant effect on standing balance in people with diabetes and neuropathy. That is, there was no statistically significant difference in standing balance when the flat (high resilient, fast release 6 mm poron cover) and no insole conditions were compared. Furthermore we concluded from the comparison diabetic (high resilient, fast return, 6 mm poron cover) and memory (low resilient, slow release, 6 mm Memory V9 cover) insole conditions, that material resilience has no statistically significant effect on standing balance in people with diabetes and neuropathy. Similarly, Van Geffen and colleagues compared the body sway of 30 people with diabetes and neuropathy standing with feet 2.5 cm together whilst wearing no insoles, 8 mm soft 15 Shore A insoles and 8 mm firm 30 Shore A value insoles [10]. This group measured Route Mean Square of the anterior posterior centre of mass velocity, and likewise found no difference in sway values between each insole tested [10].

There may be a number of possibilities to explain why variations in insole firmness and resilience had no apparent effect on standing balance in people with diabetes and neuropathy. First the 9 mm thickness of the insole condition used in this study may not be sufficient to cause a balance perturbation. Second, people with neuropathy, may not be as reliant on cutaneous sensory perception to maintain postural stability as those without neuropathy. In compensation for their sensory deficits, people with neuropathy may select to employ an alternate balance strategy. Thus important information about foot position awareness generated by changes in insole material is either overridden or goes unnoticed.

### The effect of insole texture on standing balance

When the smooth topped low resilient memory insole with arch fill was covered with a textured material static balance improved by a statistically significant amount, to a similar level to that found when participants wore the flat insoles and no insoles without arch fill. We recorded a mean reduction in total COP path length of 8 % (absolute mean 62 mm) when the low resilient memory cover was replaced by the textured cover.

This study is novel in its inclusion of people with severe diabetic neuropathy. No other study has investigated the effects of textured insoles on standing balance in people with moderate and severe neuropathy. The lack of work in this area has been rationalised by the assumption that people who are seemingly unable to perceive texture would be unlikely to respond to its affect. Our study suggests that this assumption is unfounded and texture can affect postural stability even in people with severe neuropathy.

Further exploratory subgroup analysis was undertaken to investigate if the differences in the insole effect on postural stability were dependant on neuropathy severity. Participants were split into two groups; moderate and severe neuropathy. Severe neuropathy was defined as participants with a VPT threshold of more than 40 volts. The moderate neuropathic group was had a VPT threshold of between 25 and 40 volts. The findings for the moderate and severe neuropathic groups are comparable to each other and similar to that of the whole group.

The central nervous system continuously adjusts the relative contribution of the different sensory inputs (somatosensory, visual, vestibular) according to differing environmental constraints [26]. This capacity of the central nervous system enables the body to adapt to changing conditions (e.g. light verses dark) to stay upright [26]. The introduction of the textured surface (in the absence of visual cues) may have heightened the participant’s reliance on and responsiveness to plantar sensory inputs.

Several studies have demonstrated the relationship between plantar surface sensitivity and postural control. However Palluel and colleagues found that wearing spike insoles did not enhance cutaneous sensation, yet did improve postural stability in elderly people [27]. They concluded that the absence of a correlation between plantar surface sensitivity and postural control suggested that the spikes stimulated other deep receptors. We believe that this concept may provide an explanation to the counter intuitive finding of this study, suggesting that insole texture may alter the standing balance of people with neuropathy.

There are four types of mechanoreceptors in the sole of the foot. Steady indentation pressure is sensed by the merkel disks (SAI). Rapid indentation is sensed by the Ruffini corpuscles (SAII) resulting in the sensation of skin stretch. Vibration and texture are sensed by the Pacinian corpuscles (FAII). While Meissner corpuscles (FAI) sense low frequency vibrations, resulting in the sensation of gentle fluttering [28]. Our findings may be explained if we suppose that the textured material created focal points of skin stretch or indentation pressure to generate increased SAII or SAI afferent firing. Hatton and colleagues examined the effects of textured insoles in 50 older adults’ quiet standing with eyes closed and open. They found that whilst textured pyramids (with the potential to cause focal points of skin stretch) reduced ML sway with eyes closed, the concave dimples (with less potential to cause skin stretch) had no effect [29].

SAI receptors are the most sensitive to maintained indentation [30]. They are mostly are located on the borders of the sole and represent just 14.4 % of the mechanoreceptors of the foot soles in young adults [28]. The moulded profile of the textured insole used in our study could have increased surface contact with the boarders of the sole. Thus quiet standing on the textured surface could have provided the steady indentation stimulus at the key location required for afferent firing of these mechanoreceptors.

Such a response by SAI and SAII afferents if present would not correlate well to neuropathy severity as tested by light touch or vibration thresholds [31]. The SAII afferents, are least sensitive to the light touch and vibration stimulation [32] and the SAI afferents are focused in the plantar boarders, a location not included in our neuropathy testing.

When considering the clinical relevance of these results there are a number of important limitations integral to the design of this pilot study that must be taken into account. First, changes in postural sway with the different insoles conditions in quiet standing individuals may not indicate that the insole components will have a clinical impact on every day balance activities. Second, this study focused on eyes closed conditions with feet close together, work is needed to explore whether the insole components have a similar effect on balance control under eyes open conditions. Third, the results of the study only reflect the immediate effect of the insoles tested on standing balance; the longer term effect of the insoles conditions on balance was not tested. Clinicians involved with the care of the diabetic foot should continue with current best practice; to provide those people with diabetes at increased risk of foot ulcer with an offloading insole. However, clinicians should carefully weigh up the offloading effectiveness of insoles against the possible impact of a particular insole design on balance. Especially when instability is cited by an individual as the underlying cause for insole non-adherence and an appropriate alternative offloading insole design could be provided.

## Conclusion

Insoles have an effect on static balance but not stepping reaction time. This effect is independent of neuropathy severity. The addition of a textured cover seems to counter the negative effect of an arch fill, even in participants with severe sensation loss. Static balance is unaffected by material softness or resilience. These results suggest that insole design may artificially alter the somatosensory awareness that contributes to the maintenance of postural stability in people with DPN. However variations in insole design may not influence the reaction time of any rapid voluntary corrective balance response. Current best practice is to provide offloading insoles, with arch fill, to increase contact area and reduce peak pressure and ulcer risk. The results suggest that such insoles could be making people more unstable. Instead, people with DPN who display poor postural balance when wearing standard offloading insoles, may benefit from a flat, soft insole less likely to compromise postural balance. The addition of a textured cover countered the negative effect of the arch fill. Textured insoles may alter sensory awareness even in people with DPN. The effect of texture on balance, gait and falls in people with DPN merits further investigation. There is a clear need to develop an offloading insole that can reduce diabetic foot ulcer risk, without compromising balance.

## Abbreviations

AP: Anterior/posterior
COP: Centre of pressure
DPN: Diabetic Peripheral Neuropathy
EVA: Ethylene vinyl acetate
IQR: Interquartile range
ML: Medial/lateral
SD: Standard deviation
V: Volts
